# COVID-19 vaccine acceptance among healthcare workers in India: Results from a cross-sectional survey

**DOI:** 10.1371/journal.pgph.0000661

**Published:** 2022-07-06

**Authors:** Kayur Mehta, Baldeep K. Dhaliwal, Sanjay Zodpey, Stacie Loisate, Preetika Banerjee, Paramita Sengupta, Madhu Gupta, Anita Shet

**Affiliations:** 1 Maternal and Child Health Center India, Johns Hopkins Bloomberg School of Public Health, Baltimore, Maryland, United States of America; 2 International Vaccine Access Center, Johns Hopkins Bloomberg School of Public Health, Baltimore, Maryland, United States of America; 3 Public Health Foundation of India, New Delhi, India; 4 Department of Epidemiology, Johns Hopkins Bloomberg School of Public Health, Baltimore, Maryland, United States of America; 5 Department of Community Medicine and Family Medicine, All India Institute of Medical Sciences, Kalyani, West Bengal, India; 6 Department of Community Medicine and School of Public Health, Postgraduate Institute of Medical Education and Research, Chandigarh, India; LSHTM: London School of Hygiene & Tropical Medicine, UNITED KINGDOM

## Abstract

Remarkable scientific progress has enabled expeditious development of effective vaccines against COVID-19. While healthcare workers (HCWs) have been at the frontline of the pandemic response, vaccine acceptance amongst them needs further study. We conducted a web-based survey to assess vaccine acceptance among HCWs in India between January and February 2021, shortly after the launch of India’s vaccination campaign. Descriptive statistics were used to examine respondent demographics and Likert scale responses. Binomial logistic regression analyses were used to identify factors associated with vaccine acceptance. The survey yielded 624 respondents from 25 states and five union territories in India; 53.5% were male, and median age was 37 years (IQR 32–46). Amongst all respondents, 84.1% (525/624) supported COVID-19 vaccines, and 63.2% (141/223) of those unvaccinated at the time of survey administration were willing to accept a vaccine. Trust in government sources, healthcare providers or scientific journal articles for COVID-19 related information was reported by 66.8%, while confidence in social media for this information was reported by only 4.5%. Amongst those who had not yet received a COVID-19 vaccine, factors independently associated with vaccine acceptance included age (aOR 3.50 [95% CI, 1.04–11.76] for those above 45 years compared to younger HCWs aged 18–29 years), belief in vaccine effectiveness and safety (aOR 3.78 [95% CI 1.15–12.38]), and provision of free/no-cost vaccine (aOR 2.63 [95% CI, 1.06–6.50]). Most respondents (80%) were confident about their hospital being equipped to efficiently rollout COVID-19 vaccines to the general population. While overall attitudes towards COVID-19 vaccination were positive among HCWs in India, acceptance was lower among healthier and younger HCWs. Data availability on vaccine safety and effectiveness, and cost considerations were important for acceptance. Targeted interventions are needed to improve vaccine acceptance amongst HCWs, since they are critical in promoting vaccine acceptance amongst the general population.

## Background

After first emerging in late 2019 [1], the SARS-CoV-2 virus which causes COVID-19 has spread across the world and achieved pandemic status in March 2020. In the absence of pharmaceutical interventions, population-wide lockdowns and social distancing measures were enforced to slow the spread of the virus and reduce deaths [2]. However, rapidly developing an efficacious vaccine was crucial to the control and mitigation of the pandemic. Unprecedented scientific endeavors saw the development of vaccines against SARS-CoV-2 at extraordinary speed, with clinical trials beginning within two months of the discovery of the SARS-CoV-2 genetic sequence [3]. By early January 2021, India’s drug regulator provided emergency use authorization to two vaccine candidates, one manufactured by Serum Institute of India under license from Astra Zeneca, and the other, an inactivated SARS-CoV-2 vaccine manufactured by Bharat Biotech in collaboration with Indian Council of Medical Research [4]. Due to their enhanced risk of exposure and infection, healthcare and frontline workers were prioritized for vaccination.

Understanding facilitators and barriers to vaccine uptake is critical to ensure the success of any vaccination campaign. Following the launch of the COVID-19 vaccination campaign in India, vaccine refusal among healthcare and frontline workers, despite their prioritization, was contributing to low uptake; shortly after the vaccine was authorized, only 56% of eligible HCWs had decided to accept a vaccine [5]. Studies from across the world have shown in the context of a COVID-19 vaccine, vaccine hesitancy amongst healthcare workers (HCWs) ranged from 27.7–78.1% [6]. Safety concerns, potential adverse events, and speed of vaccine development have been identified as major concerns [6].

HCWs are important sources of information for vaccines, and effective communication from HCWs with patients has been shown to improve adherence to vaccination recommendations [7,8]. Studies have shown that HCWs who are vaccinated are more likely to recommend vaccines to friends, family, and their patients [9,10]. Data regarding COVID-19 vaccine acceptance amongst HCWs in India are scarce. This study aimed to evaluate barriers to acceptance to advocate for vaccine uptake among healthcare and frontline workers in India.

## Methods

### Survey questionnaire and design

A survey to assess perceptions of HCWs towards COVID-19 vaccination was developed by researchers at the Johns Hopkins Maternal and Child Health Center in India and the Public Health Foundation of India, using vaccine hesitancy questionnaire guidelines developed by the Strategic Advisory Group of Experts on Immunization (SAGE) Working Group of the World Health Organization (WHO) that established a model of determinants of vaccine hesitancy based on systematic reviews of literature and key informant interviews [11]. The survey also drew elements from the Kaiser Family Foundation (KFF) survey that was designed to assess perceptions of the COVID-19 vaccine [12]. The questions and domains were written to be aligned with our research questions and objectives of capturing cross-sectional perceptions on vaccine acceptance from HCWs at the time of vaccine roll out within the Indian context. The survey consisted of 34 questions to document demographic information, knowledge and attitudes about COVID-19 and COVID-19 vaccines, perceptions of risk in their work setting, intent to accept a COVID-19 vaccine, and readiness among healthcare facilities to scale up vaccine delivery. Participants were asked about whether their decision to accept the COVID-19 vaccine themselves would depend on whether the vaccine was recommended by the Ministry of Health/WHO/other global agencies, whether there was favorable discussion around the vaccine in media sources, whether their peers accepted the vaccine, whether the vaccine was provided free of cost, whether there was reliable evidence that the vaccine was effective, whether the vaccine had no serious side effects, and whether they found it convenient to get themselves vaccinated. Participants could skip any questions they did not wish to answer. Vaccine hesitancy was defined as “a delay in acceptance or refusal of vaccination despite availability of vaccination services”, as per the WHO Strategic Advisory Group on Experts (SAGE) on Immunization’s definition of vaccine hesitancy [13]. All those who were not hesitant, based on this definition by the WHO, were considered as accepting of the vaccine.

### Participants and survey dissemination

The survey was administered online using the software platform Qualtrics (Qualtrics, Provo, UT) between 24 January 2021 and 28 February 2021. HCWs working in various settings in India were invited to participate after distributing the survey via professional networks such as the Public Health Foundation of India and the Indian Association of Preventive and Social Medicine. The survey was further distributed among administrative leaders across hospitals affiliated with medical colleges and among employees of other major hospital systems in India. Dissemination of the survey was done via email, text messages and social media platforms such as WhatsApp. A snowball sampling method was utilized to increase the reach of the survey. HCWs were classified on the basis of whether or not they worked in a health-facility setting, and those working in health-facility settings were further classified as patient-facing and non-patient facing. Nurses, medical doctors, clinic workers and hospital paramedical workers were considered as patient-facing; non-patient facing HCWs were reception clerks, housekeeping staff, laboratory personnel and other non-healthcare frontline workers. HCWs working as public health professionals outside of health-facility settings were classified as working in non-health-facility settings.

### Statistical analysis

Descriptive statistics, expressed as frequencies and proportions, were used to examine respondent demographics and Likert scale responses. Proportions were calculated using denominators reflective of the number of respondents for the specific question. Multiple imputation technique was used to correct for missing data in the Likert scale responses. Chi square tests were used to compare the characteristics of respondents who were unwilling to accept a COVID-19 vaccine with those who were willing to, amongst those respondents who had not received a COVID-19 vaccine at the time of the survey. Binomial logistic regression analyses were used to determine factors associated with vaccine acceptance amongst those respondents who reported that they had not yet received any COVID-19 vaccines. These analyses were restricted to those who had not yet received a COVID-19 vaccine at the time of survey administration since the questionnaires that were used to design the study tool were intended to assess vaccine acceptance and hesitancy amongst vaccine-naïve populations. Specifically, models were developed to identify characteristics associated with responses of “yes” (suggestive of vaccine acceptance) versus “no” or “I don’t know” (as a composite variable, suggestive of vaccine hesitancy) to whether or not they would be willing to accept a COVID-19 vaccine, thus suggestive of vaccine acceptance. Age was treated as a categorical variable in the regression models; age categories chosen were indicative of age groups prioritized for vaccination by the government of India. For the multivariable models, we considered variables which were statistically significant on univariable analyses, and, *a priori*, included plausible confounders based on literature. Model selection was guided using the Akaike Information Criteria (AIC), using backward selection. All analyses were conducted using the statistical package R (R Foundation for Statistical Computing, Vienna, Austria. http://www.R-project.org/).

### Ethical considerations

The study was reviewed by the Institutional Ethics Committee at the Public Health Foundation of India, and it received full approval in accordance with Indian Council of Medical research guidelines and Good Clinical Practice recommendations. The study was also reviewed by the Johns Hopkins Bloomberg School of Public Health (JHSPH) Institutional Review Board (IRB), which determined it to be exempt from human subjects research oversight. Consent to participate in the survey was provided in the introductory portion of the online survey. Participants were informed that responding to the survey request was voluntary; data obtained for the survey analysis would remain anonymous, and that completing the survey and submitting it implied that they had consented to participate in the study.

## Results

The online survey was completed by 632 respondents. Amongst these respondents, eight did not respond to the question on intent to be vaccinated which was the primary focus of this survey; following their exclusion, we had a total of 624 respondents, which is the number included in subsequent analyses in this report. These respondents were distributed across 25 states and five union territories in India (S1 Table); 53.5% were male, and the median age was 37 years (IQR 32–46). Amongst the cumulative 624 respondents, 21.5% already had a confirmed COVID-19 diagnosis in the past, and 24.7% reported having underlying medical co-morbidities which would place them at higher risk for severe COVID-19 disease (Table 1).

**Table 1:** Respondent demographics (n = 624).

Characteristics	Frequency N (%)
**Age (years)**	
Median (IQR)	37 (32–46)
Range	23–81
**Sex** Male Female Did not answer	334 (53.5) 285 (45.7) 5 (0.8)
**Type of institution** Private sector Public sector Did not answer	249 (39.9) 370 (59.3) 5 (0.8)
**Occupation** ^a^ Health-facility setting Patient facing Non-patient facing Non-health-facility setting Did not answer	469 (75.2) 437 (93.2) 32 (6.8) 150 (24) 5 (0.8)
**Average number of patients seen per week** ^b^ None <100 101–250 250–500 >500 Did not answer	71 (15.1) 250 (53.3) 90 (19.2) 38 (8.1) 17 (3.6) 3 (0.6)
**Average number of COVID-positive patients seen per week**^b^ None 1–25 26–50 >50 I don’t know Did not answer	142 (30.3) 228 (48.6) 27 (5.7) 35 (7.5) 36 (7.7) 1 (0.2)
**Underlying conditions** ^c^ Yes No Did not answer	154 (24.7) 464 (74.4) 6 (0.9)
**Have you already received one of the COVID-19 vaccines** Yes No Did not answer	401(64.3) 223 (35.7) 0 (0)
**Brand of vaccine received** ^d^ Covishield Covaxin Did not answer	365 (91) 34 (8.5) 2 (0.5)
**Previous confirmed or suspected COVID diagnosis** Yes No I don’t know Did not answer	134 (21.5) 439 (70.3) 0 (0) 51 (8.2)

^**a**^ Health-facility setting occupations that are (1) patient-facing: Nurses, medical doctors, clinic workers, hospital paramedical workers and (2) non-patient facing: Admission/reception, housekeeping/cleaning staff, laboratory personnel, non-healthcare frontline workers. Non-health-facility setting occupations are those where respondents worked in public health capacities outside of health-facility settings.

^**b**^ Among respondents who reported having an occupation in a health-facility setting **(n = 469)**.

^**c**^ Underlying conditions among respondents reporting “Yes” include one or more of the following: Asthma, cardiovascular disease, chronic lung disease, chronic renal disease, diabetes mellitus, and hypertension **(n = 154).**

^**d**^ Among respondents who reported having received at least the first dose vaccine at the time of survey completion **(n = 401)**.

Among respondents, 95.0% felt confident regarding protecting themselves from SARS-CoV-2 infection, including access to recommended personal protective equipment (PPE) (87.5%) and updated information related to COVID-19 (86.4%). Reliability on government sources, healthcare providers or scientific journal articles for COVID-19 related information was reported by 66.8%, while confidence in social media for this information was reported by only 4.5%.

### COVID-19 vaccine acceptance

Among all respondents included in the analysis, 84.1% (525/624) were accepting of the COVID-19 vaccines currently approved by the national government. An approved COVID-19 vaccine was received by 64.3% (401/624) at the time of administration of the survey. Amongst the 223 respondents who had not yet received a COVID-19 vaccine, 63.2% (141/223) were willing to accept an approved COVID-19 vaccine. Amongst these 223 respondents, those who were unwilling to accept a vaccine were younger (median age, 32 vs. 36 years, p<0.05) (statistically significant), more often had a previously confirmed or suspected COVID-19 diagnosis (25.7% vs 19.2%, p = 0.28), were less likely to have underlying conditions (20.7% vs 25.2%, p = 0.45), and fewer perceived a high degree of susceptibility to COVID-19 (19.5 vs 29.1%, p = 0.08) compared to those who were willing to accept a COVID-19 vaccine (all of which were not statistically significant) (S2 Table). No statistically significant differences were found amongst those HCWs who reported “no” or “I don’t know” to willingness to accept a COVID-19 vaccine at the time of survey administration (S3 Table).

### Factors influencing the decision to receive a COVID-19 vaccine

Eighty-three percent (518/624) of respondents believed that a vaccine would help control the spread of COVID-19. Further, 89.7% (560/624) felt that it would still be important to receive a COVID-19 vaccine even though cases were declining in India at the time of administration of the survey, prior to the second surge. Amongst respondents who had not yet received a COVID-19 vaccine at the time of survey administration, 50% (95/190) suggested that they would prefer to wait for a few months before taking a novel vaccine, 50% (111/223) suggested that their decision to accept the vaccine would be influenced by the prevalence of infection at the time of vaccination, and 71% (159/223) suggested that their decision would be influenced by the side effects of the vaccine. After adjusting for differences in participant characteristics, using multivariable logistic regression analyses, the factors independently associated with an increased likelihood of accepting the vaccine amongst those who had not yet received a COVID-19 vaccine at the time of survey administration were, advancing age (aOR 3.50 [95% CI, 1.04–11.76] for those aged above 45 years), availability of evidence that the vaccine was effective (aOR 3.78 95% CI 1.15–12.38]), and provision of a vaccine free of cost (aOR 2.63 [95% CI, 1.06–6.50]). Other demographic factors such as age, gender, occupation (health-facility vs. non-health-facility setting), type of institution where they worked (public vs. private), presence of underlying comorbidities and perceived degree of susceptibility to COVID-19 were not associated with vaccine acceptance (Table 2). Further, the respondents’ decisions to accept a COVID-19 vaccine were not significantly associated with factors such as recommendations by the ministries of health or global organizations, vaccine-related discussions on media and social media, their peers’ acceptance of the vaccine, and reported vaccine side effects (Table 2). On a multinomial logistic regression model, factors associated with vaccine acceptance included evidence of vaccine effectiveness and safety, and provision of free/no-cost vaccine (S4 Table).

**Table 2:** Factors influencing the decision to accept a COVID-19 vaccine currently approved by the government among respondents who had not yet received a vaccine at the time of survey administration (n = 223).

Characteristics	Vaccine acceptance			aOR (95% CI)
	Yes n (%)	No n (%)	I don’t know n (%)	Vaccine Acceptance: Yes versus No/I don’t know
**Age in years (n = 210)**				
18–29 (Reference)	32 (54.2)	9 (15.3)	18 (30.5)	
30–44	57 (60.6)	11 (11.7)	26 (27.7)	1.81 (0.70–4.70)
45+	42 (73.7)	4 (7.0)	11 (19.3)	3.50 (1.04–11.76)
**Gender (n = 220)**				
Female (Reference)	71 (60.2)	12 (10.2)	35 (29.7)	
Male	67 (65.7)	13 (12.7)	22 (21.6)	1.44 (0.63–3.29)
**Occupation (n = 223)**				
Non-health-facility settings (Reference)	41 (57.7)	8 (11.3)	22 (31.0)	
Health-facility settings	100 (66.7)	16 (10.7)	34 (22.7)	1.68 (0.73–3.88)
**Institution (n = 220)**				
Private sector institution (Reference)	86 (66.7)	11 (8.5)	32 (24.8)	
Public sector institution	53 (58.2)	14 (15.4)	24 (26.4)	0.83 (0.35–1.98)
**Presence of underlying conditions (n = 221)**				
Yes (Reference = no)	35 (67.3)	6 (11.5)	11 (21.2)	1.16 (0.40–3.36)
**Previous infection with SARS-CoV-2 (n = 199)**				
Yes (Reference = no)	24 (55.8)	6 (14.0)	13 (30.2)	0.44 (0.16–1.21)
**How susceptible do you consider yourself to an infection with COVID-19 (n = 223)**				
High degree of susceptibility (reference)	41 (71.9)	4 (7.0)	12 (21.1)	
Moderate degree of susceptibility	65 (65.0)	10 (10.0)	25 (25.0)	1.12 (0.40–3.13)
Low degree of susceptibility	35 (53.0)	11 (16.7)	20 (30.3)	0.56 (0.18–1.68)
**The vaccine is recommended by the Ministry of Health/WHO/other/global agencies† (n = 203)**	120 (70.6)	13 (7.6)	37 (21.8)	2.82 (0.89–8.87)
**There is favorable discussion around the vaccine in media (television, radio, print and social media) † (n = 185)**	41 (64.1)	9 (14.1)	14 (21.9)	0.75 (0.29–1.96)
**My peers accept the vaccine† (n = 190)**	63 (69.2)	6 (6.6)	22 (24.2)	1.01 (0.42–2.46)
**The vaccine has no serious side effects † (n = 197)**	87 (61.7)	12 (8.5)	42 (29.8)	0.46 (0.15–1.38)
**There is reliable evidence that the vaccine is effective† (n = 196)**	108 (67.1)	11 (6.8)	42 (26.1)	3.78 (1.15–12.38)
**It is convenient it is to get the vaccine† (n = 195)**	51 (69.9)	6 (8.2)	16 (21.9)	1.42 (0.57–3.53)
**The vaccine is free of cost† (n = 184)**	57 (75.0)	5 (6.6)	14 (18.4)	2.63 (1.06–6.50)

^**†**^The distribution shown in the table above is reflective of those responding “yes” to each of these questions, and the reference category was “no/I don’t know” for the purposes of regression analyses as discussed in the methods section. The n’s displayed in parentheses reflect the total number of respondents who answered these questions.

### Perceptions surrounding vaccine preparedness in healthcare facilities

Overall, most respondents were confident about healthcare systems being equipped to efficiently rollout COVID-19 vaccines to the general population. Confidence in different domains was reported by respondents (sufficient number of trained staff: 81.0%; procuring adequate supplies: 77.0%; efficient storage and deployment: 79.6%).

## Discussion

In a representative sample of HCWs across India, our study conducted a week into the launch of India’s COVID-19 vaccination campaign found that overall vaccine acceptance was high at 84%, but amongst those who had not yet received the vaccine at the time of survey administration, vaccine acceptance was 63%. Most respondents felt adequately empowered to find appropriate information about the disease and vaccine, indicated having knowledge on infection prevention, and believed that a vaccine would help in curtailing the spread of the infection. HCWs were more likely to accept the vaccine if they were older, had access to reliable evidence of vaccine effectiveness, and were provided with vaccine that was free of cost.

Being arbiters of scientific and public health information, HCWs are critical in promoting vaccine acceptance. As vaccine recipients themselves, and vaccine advocates, they can play a pivotal role in helping with vaccine messaging, addressing vaccine hesitancy among the public, and promoting rapid vaccine coverage within countries and globally. Vaccinating HCWs is also particularly important, as they are at increased risk of infection acquisition due to the nature of their work. In a recent systematic review on COVID-19 vaccine hesitancy worldwide, which included a total of 31 studies, eight studies were pertinent to HCWs; it was found that vaccine acceptance rates ranged from 27.7% in the Democratic Republic of the Congo to 78.1% in Israel [6]. A nationwide survey of undergraduate medical students in India conducted at the launch of the vaccination campaign in India found that 89.2% participants were willing to accept a vaccine [14]. In our study, we found that amongst the HCWs who had not yet received a COVID-19 vaccine, acceptance was at 63%. Studies of vaccine acceptance amongst HCWs have shown that factors associated with acceptance include male sex [15,16], older age [16–18], education level [17,18], perceiving a high risk for infection [15], friends/peers/relatives having had previous COVID-19 infection [15,19], trust with their government’s measures [15] and occupational exposure [16]. Recent data have also suggested that individuals were influenced by the effectiveness of COVID-19 vaccines, and they would be less willing to accept vaccines with low efficacy [20,21].

Cost considerations were important as was found in our study. Affordability has often been cited as a barrier to vaccine uptake [22]. India’s income inequality ranks among the highest in the world and it is largely due to regional disparities, rich-poor divide and the urban-rural gap [23]. As the federal government in India has decided to make COVID-19 vaccines available free of cost to all adults beginning June 21, 2021, it is possible that this will continue to motivate individuals to accept the COVID-19 vaccines, and improve the equity in accessing vaccines [24]. Following this change, data indicate that the average number of daily doses administered have exceeded those documented in earlier periods, and these are encouraging signs [25].

Additionally, our survey demonstrated that nearly 50% of respondents’ decisions to accept a COVID-19 vaccine would be impacted by the prevalence of COVID-19 in India at the time they were offered a vaccine. India’s recent surge of cases and deaths during the second wave of COVID-19 cases, partially attributed to the increase in viral variants [26], highlights the need for vaccination programs to outpace the development of viral variants. Scaling up vaccine manufacturing and rolling out vaccines as quickly and widely as possible are critical to protect citizens before they are exposed to the virus and new variants [27]. Achieving a high vaccine coverage level to reach herd immunity is also critical in combatting the delta variant, which is on the rise and is the most infectious variant known so far [28], and largely responsible for the second surge of COVID-19 cases in India, as well as outbreaks across the world [29].

While identifying factors associated with acceptance is critical for public health messaging, identifying factors that contribute to hesitancy is similarly critical to ending the pandemic. Although HCWs in the United States have acknowledged the importance of vaccination to public health, many still express apprehensions over serious adverse effects from the COVID-19 vaccine [30]. In our study, a majority of respondents shared that their decision to accept a vaccine would depend on whether the vaccine does not have serious side effects; however, this did not achieve statistical significance when analyzed in the subgroup of HCWs who had not yet received the vaccine. Skepticism over the fast-tracked vaccine development and approval process has also contributed to hesitancy amongst HCWs worldwide [16,31,32]. In our study, 50% of HCWs stated that their decision to accept a vaccine would be influenced by whether the vaccine has been in use for over six months.

It is critical to effectively design communication strategies to address the unique concerns HCWs and general populations may have about the COVID-19 vaccine. Vaccinated HCWs have a noticeable positive impact on individuals’ decisions to accept a COVID-19 vaccine [33]; therefore, messaging that emphasizes that majority of vaccination experiences are positive, and highlights acceptance among HCWs, will lead to more community support of COVID-19 vaccines. Communication campaigns need to leverage strategies to design community-informed social and behavioral messages, target positive factors associated with acceptance [34], and address factors that impact hesitancy. Efforts that put people at the center of efforts may culminate in increased vaccine uptake [35]. This is particularly critical to consider as India is currently in the process of scaling up programs to provide all adults with an additional booster dose of the COVID-19 vaccine [36]. India has successfully vaccinated 73% of its 18+ population with their first dose of the COVID-19 vaccine, roughly 61% of the 18+ population with both doses, and only 1.7% of the 18+ population with an additional booster dose [37]. In light of this decline between first and second doses and limited uptake of boosters, leveraging HCWs acceptance of COVID-19 vaccines, and harnessing the advocacy of HCWs, as identified by our research, may be essential to expanding COVID-19 vaccine uptake of second doses, and particularly boosters, across India.

Accessible and equitable global roll-out of vaccines for COVID-19 is essential to curb the spread of the pandemic, protect against severe disease, and drive herd immunity. According to the World Health Organization’s COVID-19 vaccine introduction toolkit [38], successful deployment of the vaccines will involve regulatory preparedness, identifying target populations and delivery strategies, handling supply and related logistics, adequate human resources and training and providing vaccine specific resources. Most respondents in our study felt that health facilities in India were adequately prepared to undertake the massive endeavor of the largest vaccination drive ever attempted by any country. Since the launch of the COVID-19 vaccination campaign in India on January 16, 2021, over 50% of the population have received at least one dose, and 24.4% are fully vaccinated as of October 31, 2021 [39,40]. It will remain critical to sustain and scale up these efforts as the vaccination campaign advances in order to successfully control the spread of the pandemic.

Our study has some limitations that are worth noting. The survey was initiated one week after the initial rollout of the COVID-19 vaccines in India when information, options, and perceptions were rapidly changing. We acknowledge the possibility of selection bias; those who were respondents to our survey may have had strong opinions, whether positive or negative, about vaccination. Selection bias may have also arisen because the survey was administered in English on an internet supported platform; non-English speaking HCWs and those without access to the internet would not have been able to participate in this survey. Responses by the HCWs may have been affected by social desirability bias. Further, this was a cross-sectional survey administered at a single time-point, with no longitudinal follow-up; willingness to get vaccinated may change over time and be influenced by factors such as newer knowledge and the emergence of variants. We did not assess acceptance towards the second or subsequent doses of the vaccine as the vaccine rollout had just begun, and most vaccine recipients had received a single vaccine dose at the time of the survey. This survey was conducted during a time when COVID-19 cases were on the decline in India, prior to the second surge, which could have impacted perceptions of individuals responding to our survey. The cross-sectional survey design was employed to understand HCWs’ perspectives towards COVID-19 vaccination, the rapid rollout of which precluded us from strengthening our sampling methodology. As a result, the results of this survey should be interpreted in context, as they may not necessarily represent the viewpoints of all HCWs in India. Despite these limitations, our cross-sectional study is strengthened by the fact that we received responses from a diverse group of HCWs from across India.

In conclusion, in this cross-sectional survey conducted at the onset of the COVID-19 vaccination rollout in India, we found that amongst a representative group of HCWs in India, overall attitudes towards COVID-19 vaccination were positive, with the majority willing to accept a vaccine. Favorable vaccine efficacy and provision of vaccination free of cost were significantly associated with acceptance. As HCWs serve as ambassadors for evidence-based medical interventions, and they are critical in promoting vaccine acceptance amongst the general population, it is essential to design targeted interventions to improve vaccine acceptance amongst this population. Successful rollout of COVID-19 vaccines in an effort to mitigate the effects of the pandemic will require leveraging public health allies to improve trust and confidence in communities across the country—an endeavor that needs to start with healthcare workers. Learnings from this study are applicable to vaccine acceptance amongst HCWs beyond the setting of COVID-19 as well, as factors associated with vaccine acceptance are likely to be consistent across various disease settings.

## Supporting information

S1 Table Geographic location of the respondents of this survey (n = 624). (DOCX)

S2 Table Comparison of HCWs by willingness to accept a COVID-19 vaccine, amongst those who had not received a vaccine at the time of survey administration (n = 223). (DOCX)

S3 Table Comparison of HCWs who reported “no” or “I don’t know” to willingness to accept a COVID-19 vaccine at the time of survey administration (n = 82). (DOCX)

S4 Table Multinomial analysis evaluating factors influencing the decision to accept a COVID-19 vaccine (Yes vs No and I don’t know vs No) currently approved by the government among respondents who had not yet received a vaccine at the time of survey administration (n = 223). (DOCX)

S1 Data Study questionnaire. (DOCX)

S2 Data Dataset. (XLSX)
