# Supplementary material for: COVID-19 vaccine acceptance among healthcare workers in India: Results from a cross-sectional survey
Source: PLOS Glob Public Health. 2022 Jul 6;2(7):e0000661. doi: 10.1371/journal.pgph.0000661 (PMC10021553; doi:10.1371/journal.pgph.0000661)
Supplement: S1 Table — (DOCX) [file pgph.0000661.s001.docx]

**S1 Table.** Geographic location of the respondents of this survey (n=624)

| **State/ Union Territory** | **n (%)** |
| --- | --- |
| Maharashtra | 87 (13.9) |
| Karnataka | 71 (11.3) |
| Chhattisgarh | 61 (9.7) |
| Haryana | 41 (6.5) |
| Gujarat | 38 (6.0) |
| Uttar Pradesh | 28 (4.4) |
| Delhi (national capital region) | 26 (4.1) |
| Meghalaya | 24 (3.8) |
| Kerala | 24 (3.8) |
| Jharkhand | 24 (3.8) |
| Rajasthan | 18 (2.8) |
| Jammu and Kashmir | 16 (2.5) |
| Andhra Pradesh | 15 (2.4) |
| West Bengal | 15 (2.4) |
| Telangana | 14 (2.2) |
| Nagaland | 13 (2.1) |
| Assam | 12 (1.9) |
| Madhya Pradesh | 12 (1.9) |
| Odisha | 11 (1.7) |
| Tamil Nadu | 10 (1.6) |
| Bihar | 7 (1.1) |
| Arunachal Pradesh | 5 (0.8) |
| Punjab | 4 (0.6) |
| Chandigarh | 3 (0.5) |
| Himachal Pradesh | 2 (0.3) |
| Tripura | 1 (0.1) |
| Goa | 1 (0.1) |
| Puducherry (former Pondicherry) | 1 (0.1) |
| Andaman and Nicobar Islands | 1 (0.1) |
| Did not respond | 39 (6.2) |
