# Supplementary material for: COVID-19 vaccine acceptance among healthcare workers in India: Results from a cross-sectional survey
Source: PLOS Glob Public Health. 2022 Jul 6;2(7):e0000661. doi: 10.1371/journal.pgph.0000661 (PMC10021553; doi:10.1371/journal.pgph.0000661)
Supplement: S2 Table — (DOCX) [file pgph.0000661.s002.docx]

**S2 Table**. Comparison of HCWs by willingness to accept a COVID-19 vaccine, amongst those who had not received a vaccine at the time of survey administration (n=223)

| **Characteristics** | **Total**  **(n=223)** | **Yes (willing to accept a vaccine)**  **n = 141** | **No/I don’t know (unwilling to accept a vaccine)**  **n = 82** | **p-value** |
| --- | --- | --- | --- | --- |
| **Median age (IQR) (years)** | 35 (29-46) | 36 (30-49) | 32 (27-42) | 0.02 |
| **Gender** |  |  |  |  |
| Male | 102 (46.4) | 67 (48.6) | 35 (42.7) | 0.39 |
| Female | 118 (53.6) | 71 (51.4) | 47 (57.3) |  |
|  |  |  |  |  |
| **Type of institution** |  |  |  |  |
| Private sector | 129 (58.6) | 86 (61.9) | 43 (53.1) | 0.20 |
| Public sector | 91 (41.4) | 53 (38.1) | 38 (46.9) |  |
|  |  |  |  |  |
| **Occupation ^a^** |  |  |  |  |
| Health-facility setting | 150 (67.9) | 100 (70.9) | 50 (62.5) | 0.19 |
| Non-health-facility setting | 71 (32.1) | 41 (29.1) | 30 (37.5) |  |
|  |  |  |  |  |
| **Presence of underlying conditions ^b^** | 52 (23.3) | 35 (25.2) | 17 (20.7) | 0.45 |
|  |  |  |  |  |
| **Previous confirmed or suspected COVID-19 diagnosis** | 43 (19.2) | 24 (19.2) | 19 (25.7) | 0.28 |
|  |  |  |  |  |
| **How susceptible do you consider yourself to an infection with COVID-19** | | | | |
| High degree of susceptibility | 57 (25.6) | 41 (29.1) | 16 (19.5) | 0.08 |
| Moderate degree of susceptibility | 100 (44.8) | 65 (46.1) | 35 (42.7) |  |
| Low degree of susceptibility | 66 (29.6) | 35 (24.8) | 31 (37.8) |  |

^a^ Health-facility setting occupations that are (1) patient-facing: nurses, medical doctors, clinic workers, hospital paramedical workers and (2) non-patient facing: admission/reception, housekeeping/cleaning staff, laboratory personnel, non-healthcare frontline workers. Non-health-facility setting occupations are those where respondents worked in public health capacities outside of health-facility settings.

^b^ Underlying conditions among respondents reporting “Yes” include one or more of the following: asthma, cardiovascular disease, chronic lung disease, chronic renal disease, diabetes mellitus, and hypertension.
