# Supplementary material for: COVID-19 vaccine acceptance among healthcare workers in India: Results from a cross-sectional survey
Source: PLOS Glob Public Health. 2022 Jul 6;2(7):e0000661. doi: 10.1371/journal.pgph.0000661 (PMC10021553; doi:10.1371/journal.pgph.0000661)
Supplement: S3 Table — (DOCX) [file pgph.0000661.s003.docx]

**S3 Table**. Comparison of HCWs who reported “no” or “I don’t know” to willingness to accept a COVID-19 vaccine at the time of survey administration (n=82)

| **Characteristics** | **Total**  **(n=82)** | **No**  **n = 25** | **I don’t know**  **n = 57** | **p-value** |
| --- | --- | --- | --- | --- |
| **Median age (IQR) (years)** | 32 (27-42) | 32 (27-42) | 31 (28-41.8) | 0.46 |
| **Gender** |  |  |  |  |
| Male | 35 (42.7) | 13 (52.0) | 22 (38.6) | 0.26 |
| Female | 47 (57.3) | 12 (48.0) | 35 (61.4) |  |
|  |  |  |  |  |
| **Type of institution** |  |  |  |  |
| Private sector | 43 (53.1) | 11 (44.0) | 32 (57.1) | 0.27 |
| Public sector | 38 (46.9) | 14 (56.0) | 24 (42.9) |  |
|  |  |  |  |  |
| **Occupation ^a^** |  |  |  |  |
| Health-facility setting | 50 (62.5) | 16 (66.7) | 34 (60.7) | 0.61 |
| Non-health-facility setting | 30 (37.5) | 8 (33.3) | 22 (39.3) |  |
|  |  |  |  |  |
| **Presence of underlying conditions ^b^** | 17 (20.7) | 6 (24.0) | 11 (19.3) | 0.63 |
|  |  |  |  |  |
| **Previous confirmed or suspected COVID-19 diagnosis** | 19 (25.7) | 6 (25.0) | 13 (26.0) | 0.93 |
|  |  |  |  |  |
| **How susceptible do you consider yourself to an infection with COVID-19** | | | | |
| High degree of susceptibility | 16 (19.5) | 4 (16.0) | 12 (21.0) | 0.72 |
| Moderate degree of susceptibility | 35 (42.7) | 10 (44.0) | 20 (35.1) |  |
| Low degree of susceptibility | 31 (37.8) | 11 (40.0) | 25 (43.9) |  |

^a^ Health-facility setting occupations that are (1) patient-facing: nurses, medical doctors, clinic workers, hospital paramedical workers and (2) non-patient facing: admission/reception, housekeeping/cleaning staff, laboratory personnel, non-healthcare frontline workers. Non-health-facility setting occupations are those where respondents worked in public health capacities outside of health-facility settings.

^b^ Underlying conditions among respondents reporting “Yes” include one or more of the following: asthma, cardiovascular disease, chronic lung disease, chronic renal disease, diabetes mellitus, and hypertension.
