# Supplementary material for: COVID-19 vaccine acceptance among healthcare workers in India: Results from a cross-sectional survey
Source: PLOS Glob Public Health. 2022 Jul 6;2(7):e0000661. doi: 10.1371/journal.pgph.0000661 (PMC10021553; doi:10.1371/journal.pgph.0000661)
Supplement: S4 Table — (DOCX) [file pgph.0000661.s004.docx]

**S4 Table.** Multinomial analysis evaluating factors influencing the decision to accept a COVID-19 vaccine (Yes vs No and I don’t know vs No) currently approved by the government among respondents who had not yet received a vaccine at the time of survey administration (**n = 223)**

| **Characteristics** | **Vaccine acceptance** | | | **RRR (95% CI)** | |
| --- | --- | --- | --- | --- | --- |
|  | **Yes** | **No** | **I don’t know** | **Vaccine Acceptance:** | |
|  | n (%) | n (%) | n (%) | Yes versus No | I don’t know versus no |
| **Age in years** **(n = 210)** |  |  |  |  |  |
| 18-29 | 32 (54.2) | 9 (15.3) | 18 (30.5) | Reference | Reference |
| 30-44 | 57 (60.6) | 11 (11.7) | 26 (27.7) | 3.17 (0.56-18.04) | 1.98 (0.34-11.61) |
| 45+ | 42 (73.7) | 4 (7.0) | 11 (19.3) | 3.5 (0.39-30.96) | 1 (0.1-10.23) |
| **Gender (n = 220)** |  |  |  |  |  |
| Female | 71 (60.2) | 12 (10.2) | 35 (29.7) | Reference | Reference |
| Male | 67 (65.7) | 13 (12.7) | 22 (21.6) | 0.87 (0.19-3.93) | 0.53 (0.11-2.49) |
| **Occupation** **(n = 223)** |  |  |  |  |  |
| Non-health-facility settings | 41 (57.7) | 8 (11.3) | 22 (31.0) | Reference | Reference |
| Health-facility settings | 100 (66.7) | 16 (10.7) | 34 (22.7) | 0.74 (0.16-3.39) | 0.35 (0.07-1.65) |
| **Institution (n = 220)** |  |  |  |  |  |
| Private sector institution | 86 (66.7) | 11 (8.5) | 32 (24.8) | Reference | Reference |
| Public sector institution | 53 (58.2) | 14 (15.4) | 24 (26.4) | 1.01 (0.19-5.25) | 1.26 (0.23-6.82) |
| **Presence of underlying conditions (n = 221)** |  |  |  |  |  |
| Yes (Reference = no) | 35 (67.3) | 6 (11.5) | 11 (21.2) | 2.39 (0.32-17.95) | 2.39 (0.31-18.77) |
| **Previous infection with SARS-CoV-2 (n = 199)** |  |  |  |  |  |
| Yes (Reference = no) | 24 (55.8) | 6 (14.0) | 13 (30.2) | 0.38 (0.07-2) | 0.86 (0.16-4.59) |
| **How susceptible do you consider yourself to an infection with COVID-19 (n = 223)** |  |  |  |  |  |
| High degree of susceptibility | 41 (71.9) | 4 (7.0) | 12 (21.1) | Reference | Reference |
| Moderate degree of susceptibility | 65 (65.0) | 10 (10.0) | 25 (25.0) | 1.21 (0.18-8.32) | 1.12 (0.14-9.03) |
| Low degree of susceptibility | 35 (53.0) | 11 (16.7) | 20 (30.3) | 0.83 (0.1-7.04) | 1.63 (0.17-15.57) |
| **The vaccine is recommended by the Ministry of Health/WHO/other/global agencies (n=203)** | 120 (70.6) | 13 (7.6) | 37 (21.8) | 2.26 (0.26-19.49) | 0.74 (0.09-6.33) |
| **There is favorable discussion around the vaccine in media (television- radio- print and social media) (n=185)** | 41 (64.1) | 9 (14.1) | 14 (21.9) | 0.2 (0.03-1.14) | 0.19 (0.03-1.16) |
| **My peers accept the vaccine (n=190)** | 63 (69.2) | 6 (6.6) | 22 (24.2) | 1.3 (0.22-7.56) | 1.31 (0.21-7.96) |
| **The vaccine has no serious side effects (n=197)** | 87 (61.7) | 12 (8.5) | 42 (29.8) | 0.41 (0.05-3.19) | 0.89 (0.1-7.92) |
| **There is reliable evidence that the vaccine is effective (n=196)** | 108 (67.1) | 11 (6.8) | 42 (26.1) | 26.93 (3.06-236.85) | 13.81 (1.4-136.14) |
| **It is convenient it is to get the vaccine (n=195)** | 51 (69.9) | 6 (8.2) | 16 (21.9) | 1.11 (0.19-6.59) | 0.77 (0.12-5.07) |
| **The vaccine is free of cost (n=184)** | 57 (75.0) | 5 (6.6) | 14 (18.4) | 14.21 (1.8-111.96) | 7.52 (0.91-62.13) |
