# Supplementary material for: COVID-19 vaccine acceptance among healthcare workers in India: Results from a cross-sectional survey
Source: PLOS Glob Public Health. 2022 Jul 6;2(7):e0000661. doi: 10.1371/journal.pgph.0000661 (PMC10021553; doi:10.1371/journal.pgph.0000661)
Supplement: S1 Data — (DOCX) [file pgph.0000661.s005.docx]

**COVID-19 Vaccine Preparedness and Acceptance Among Healthcare Workers in India**

**Introduction**

In light of the unprecedented speed of COVID vaccine development and rollout, as well as questions asked by the general public, we are conducting this survey to understand health care workers’ and frontline workers’ thoughts and perceptions of the newly approved COVID-19 vaccines. Please respond to these questions in your professional capacity.

This is a quick survey and should only take you approximately 10  minutes. We will not collect your name or other personal information, and your data will not attach to your institution or organization.

Responding to this survey request is voluntary; it is your choice.  Completing this survey and submitting it would mean that you consent to participate in the study. You may choose not to answer any question that we ask.

We are grateful to you for responding to our survey.

| **Demographics** | | | | | | |
| --- | --- | --- | --- | --- | --- | --- |
| **1a** | What is your occupation? | | - Medical doctor - Nurse   - - Registered nurse     - Assistant nurse   - Community Health Worker (ASHA, Anganwadi Worker, ANM) - Public health professional (epidemiologist, surveillance team member, data collector, etc.) - Hospital paramedical workers (X-ray technician, phlebotomist, physical therapist, nutritionist) - Clinic Worker - Laboratory personnel - Admission/reception clerk - General Duty Attendant - Catering staff - Housekeeping/cleaning staff - Non-Healthcare Frontline Worker - Other   If other, specify: ________________ | | | |
| **1b** | What type of institution do you work at? | | - Private sector institution - Public sector institution | | | |
| **1c** | Will you be eligible to receive a vaccine in the initial rollout of the COVID-19 vaccines in India? | | - Yes - No - I do not know | | | |
| **1d** | Have you already received one of the COVID-19 vaccines? | | - Yes - No | | | |
| **1e** | (Skip logic If Yes to 1D) which brand of the vaccine did you receive? | | - - Covishield (AstraZeneca/Oxford/Serum Institute) vaccine   - Covaxin (Bharat Biotech) vaccine   - Other, specify: | | | |
| **2** | What is your age? | | _________ years | | | |
| **3** | What is your gender? | | - - Male   - Female | | | |
| **4** | Do you suffer from any of the following conditions (check all that apply)?: | | - - Hypertension   - Diabetes Mellitus   - Cardiovascular Disease   - Asthma   - Other, specify: _____________ | | | |
| **5** | What state or Union Territory are you based in? | | - Andhra Pradesh - Arunachal Pradesh - Assam - Bihar - Chhattisgarh - Goa - Gujarat - Haryana - Himachal Pradesh - Jammu and Kashmir - Jharkhand - Karnataka - Kerala - Madhya Pradesh - Maharashtra - Manipur - Meghalaya - Mizoram - Nagaland - Odisha (former Orissa) - Punjab - Rajasthan - Sikkim - Tamil Nadu - Tripura - Uttar Pradesh - Uttarakhand - West Bengal - Telangana - Andaman and Nicobar Islands - Chandigarh - Dadra and Nagar Haveli and Daman and Diu - Jammu and Kashmir - Ladakh - Lakshadweep - Delhi (national capital territory) - Puducherry (former Pondicherry) | | | |
| **COVID-19 Personal Experience** | | | | | | |
| **6** | To your knowledge, are you, or have you been, infected with COVID-19?  (If no/don’t know [skip to question 8]) | | ☐Yes ☐ No ☐ Don’t know | | | |
| **7** | *If yes to 6, was it confirmed by a test?* | | ☐Yes ☐ No | | | |
| **8** | *If yes to 6, were you hospitalized?* | | ☐Yes ☐ No | | | |
| **9** | Do you know people in your immediate social environment who are or have been infected with COVID-19 (suspected or confirmed)? | | ☐Yes ☐ No ☐ Don’t know | | | |
| **Knowledge & attitude** | | | | | | |
| **10** | Please indicate what resources you use to stay up to date on the latest information for the COVID-19 outbreak and COVID-19 vaccines. | | | | | |
|  | | | Currently using (tick all that apply) | | | |
|  | Radio/ television | | ☐ | | | |
|  | Friends/ Colleagues | | ☐ | | | |
|  | Hospital website | | ☐ | | | |
|  | Social media (Twitter, Whatsapp, Facebook etc) | | ☐ | | | |
|  | Government source | | ☐ | | | |
|  | Journal articles | | ☐ | | | |
|  | Newspaper/Magazines | | ☐ | | | |
|  | Family members | | ☐ | | | |
|  | Community members / Friends | | ☐ | | | |
|  | Healthcare providers | | ☐ | | | |
|  | I don’t keep up to date | | ☐ | | | |
|  | Internet | | ☐ | | | |
|  | Other | | ☐ | | | |
|  | If other, please specify | |  | | | |
| **11A** | Amongst options listed above, which do you think is the ***most*** reliable? | _____ | **11B** | Amongst options listed above, which do you think is the ***least*** reliable ? | | _____ |
|  | *TICK the correct response for all Likert scale questions:* | | | | | |
| **12** | I find it easy to find the information I need related to COVID-19 | | ☐ Strongly Agree  ☐ Agree  ☐ Neither agree nor disagree  ☐ Disagree  ☐ Strongly Disagree | | | |
| **COVID-19 Risk Perception** | | | | | | |
| **13a** | Overall, how many patients do you see or interact with per week on average? | | | | ☐ None  ☐ Less than 100  ☐ 101 – 250  ☐ 250 – 500  ☐ More than 500 | |
| **13b** | How many COVID patients do you see or interact with per week on average? | | | | ☐ None  ☐ 1 - 25  ☐ 26 - 50  ☐ 51 - 75  ☐ 76 - 100  ☐ More than 100  ☐ I do not know | |
| **14** | How susceptible do you consider yourself to an infection with COVID-19? | | | | ☐ Not at all susceptible  ☐ Not very susceptible  ☐ Somewhat susceptible  ☐ Very susceptible  ☐ Extremely susceptible | |
| **Preparedness and perceived self-efficacy: Self-assessed COVID-19 self-protection and avoidance ability**  *Next, we would like to know about your own practices related to COVID-19.* | | | | | | |
| **15** | I know how to protect myself from COVID-19. | | | | ☐ Strongly agree  ☐ Agree  ☐ Neither agree nor disagree  ☐ Disagree  ☐ Strongly disagree | |
| **16** | I am able to follow infection control guidelines and use PPE as recommended. | | | | ☐ Strongly agree  ☐ Agree  ☐ Neither agree nor disagree  ☐ Disagree  ☐ Strongly disagree  ☐ N/A – I do not work in a hospital/clinic setting | |
| **COVID -19 Vaccine Acceptance**  *COVID-19 vaccines are in the initial phases of being rolled out in several countries, including India. Please share your position on the rollout of these COVID-19 vaccines:* | | | | | | |
| **17** | Now that a vaccine has been approved by the Government of India, do you think you will…? (Check one) | | | | - Receive the vaccine as soon as it is available to you - Wait until it has been available for 6 months, to see how it affects other people - Only receive the vaccine if I’m required to for work - Definitely not receive it - I do not know - Other, Specify: __________________ | |
| **18** | I believe a COVID-19 vaccine can help control the spread of COVID-19. | | | | ☐ Strongly agree  ☐ Agree  ☐ Neither agree nor disagree  ☐ Disagree  ☐ Strongly disagree | |
| **19** | Although COVID-19 cases are decreasing in India, it is still very important to receive a COVID-19 vaccine | | | | ☐ Strongly agree  ☐ Agree  ☐ Neither agree nor disagree  ☐ Disagree  ☐ Strongly disagree | |
| **20** | Do you think taking a COVID-19 vaccine is more risky than contracting COVID? | | | | - - Yes   - No   - I do not know | |
| **21** | Will you accept one of the COVID-19 vaccines currently approved by the government? | | | | - - Yes   - No   - I do not know / I would need more information | |
| **22a** | Would your decision to receive a vaccine depend on the brand of the vaccine? | | | | - - Yes, I would only accept the Covishield (AstraZeneca/Oxford/Serum Institute) vaccine   - Yes, I would only accept the Covaxin (Bharat Biotech) vaccine   - Yes, but I am waiting for a different vaccine to be approved   (Specify product _____________)   - - No, I would NOT accept any currently approved vaccine   - No, I would accept ANY approved vaccine   - N/A – I have already received a vaccine | |
| **22b** | (Skip logic: If N/A on 22a) If you have already received the vaccine, did the **brand of the vaccine** impact your decision? | | | | - - No, I was not given a choice   - No, I was willing to accept ANY approved vaccine   - Yes, I was only willing to accept the Covishield (AstraZeneca/Oxford/Serum Institute) vaccine   - Yes, I was only willing to accept the Covaxin (Bharat Biotech) vaccine   - Other, specify: | |
| **23** | If I knew I had been infected with COVID-19 before, I would not get a COVID-19 vaccine. | | | | ☐ Strongly agree  ☐ Agree  ☐ Neither agree nor disagree  ☐ Disagree  ☐ Strongly disagree | |
| **24** | If my antibody test for COVID-19 were positive, I would not get a COVID-19 vaccine | | | | ☐ Strongly agree  ☐ Agree  ☐ Neither agree nor disagree  ☐ Disagree  ☐ Strongly disagree | |
| **25** | When everyone else is vaccinated against COVID-19, then I don't have to get vaccinated. | | | | ☐ Strongly agree  ☐ Agree  ☐ Neither agree nor disagree  ☐ Disagree  ☐ Strongly disagree | |
| **26** | **My decision to receive the COVID-19 vaccine would depend on:** | | | | | |
| **26A** | *If the vaccine is produced in India* | | | | ☐ Strongly agree  ☐ Agree  ☐ Neither agree nor disagree  ☐ Disagree  ☐ Strongly disagree | |
| **26B** | *If the vaccine is produced abroad (not in India)* | | | | ☐ Strongly agree  ☐ Agree  ☐ Neither agree nor disagree  ☐ Disagree  ☐ Strongly disagree | |
| **26C** | *Recommendation of the Ministry of Health/ WHO/ other/ global agencies* | | | | ☐ Strongly agree  ☐ Agree  ☐ Neither agree nor disagree  ☐ Disagree  ☐ Strongly disagree | |
| **My decision to receive the COVID-19 vaccine would depend on:** | | | | | | |
| **26D** | *Discussion around vaccine in media (television, radio, print media) and social media* | | | | ☐ Strongly agree  ☐ Agree  ☐ Neither agree nor disagree  ☐ Disagree  ☐ Strongly disagree | |
| **26E** | *Whether the vaccine has been in use for a long time (over 6 months)* | | | | ☐ Strongly agree  ☐ Agree  ☐ Neither agree nor disagree  ☐ Disagree  ☐ Strongly disagree | |
| **26F** | *Whether my peers accept the vaccine* | | | | ☐ Strongly agree  ☐ Agree  ☐ Neither agree nor disagree  ☐ Disagree  ☐ Strongly disagree | |
| **My decision to receive the COVID-19 vaccine would depend on:** | | | | | | |
| **26G** | *Whether the vaccine has no serious side effects* | | | | ☐Strongly agree  ☐ Agree  ☐ Neither agree nor disagree  ☐ Disagree  ☐ Strongly disagree | |
| **26H** | *Reliable evidence that the vaccine is effective* | | | | ☐ Strongly agree  ☐ Agree  ☐ Neither agree nor disagree  ☐ Disagree  ☐ Strongly disagree | |
| **26I** | *High prevalence of COVID-19 at the time when vaccine is available* | | | | ☐Strongly agree  ☐ Agree  ☐ Neither agree nor disagree  ☐ Disagree  ☐ Strongly disagree | |
| **My decision to receive the COVID-19 vaccine would depend on:** | | | | | | |
| **26J** | *How convenient it is to get the vaccine (e.g. available out-of-hours or in pharmacies)* | | | | ☐Strongly agree  ☐ Agree  ☐ Neither agree nor disagree  ☐ Disagree  ☐ Strongly disagree | |
| **26K** | *Whether the vaccine is free of cost* | | | | ☐Strongly agree  ☐ Agree  ☐ Neither agree nor disagree  ☐ Disagree  ☐ Strongly disagree | |
| **26L** | *Whether hospitals and health facilities are prepared to introduce a COVID-19 vaccine* | | | | ☐Strongly agree  ☐ Agree  ☐ Neither agree nor disagree  ☐ Disagree  ☐ Strongly disagree | |
| **COVID -19 Vaccine Readiness**  *COVID-19 vaccines are in the initial phases of being rolled out in several countries, including India. Please share your thoughts on how prepared you feel your hospital is to roll out a COVID-19 vaccine.* | | | | | | |
| **27** | I believe that my hospital has a sufficient number of trained staff to roll out a COVID-19 vaccine. | | | ☐ Strongly agree  ☐ Agree  ☐ Neither agree nor disagree  ☐ Disagree  ☐ Strongly disagree  ☐ N/A – I do not work in a hospital/clinic setting | | |
| **28** | I believe that my hospital will be able to procure supplies needed to roll out a COVID-19 vaccine efficiently (ex: syringes, needles, cold storage equipment). | | | ☐ Strongly agree  ☐ Agree  ☐ Neither agree nor disagree  ☐ Disagree  ☐ Strongly disagree  ☐ N/A – I do not work in a hospital/clinic setting | | |
| **29** | My hospital has adequate facilities for vaccine storage, handling, administration, and documentation. | | | ☐ Strongly agree  ☐ Agree  ☐ Neither agree nor disagree  ☐ Disagree  ☐ Strongly disagree  ☐ N/A – I do not work in a hospital/clinic setting | | |
| **30** | I believe my hospital will be able to acquire sufficient numbers of vaccine doses to provide to HCWs eligible to receive vaccines. | | | ☐ Strongly agree  ☐ Agree  ☐ Neither agree nor disagree  ☐ Disagree  ☐ Strongly disagree  ☐ N/A – I do not work in a hospital/clinic setting | | |
| **31** | I believe that facilities are in place to maintain the cold chain for a large-scale vaccination drive. | | | ☐ Strongly agree  ☐ Agree  ☐ Neither agree nor disagree  ☐ Disagree  ☐ Strongly disagree | | |
